# Calcified brain metastases may be more frequent than normally considered

**DOI:** 10.1007/s00330-020-07164-2

**Published:** 2020-08-19

**Authors:** Giacomo Rebella, Nicola Romano, Giulia Silvestri, Jean Louis Ravetti, Gabriele Gaggero, Liliana Belgioia, Francesco Lupidi, Alessio Signori, Luca Roccatagliata, Laura Saitta, Lucio Castellan

**Affiliations:** 1 Department of Health Sciences (DISSAL), University of Genoa, Genoa, Italy; 2 Pathology Unit, Ospedale Policlinico San Martino, Genoa, Italy; 3 Department of Radiation Oncology, IRCCS Policlinico San Martino, Genoa, Italy; 4 Department of Health Sciences (DISSAL), Section of Biostatistics, University of Genoa, Genoa, Italy; 5 Department of Neuroradiology, IRCCS Ospedale Policlinico San Martino, Genoa, Italy

**Keywords:** Brain neoplasms, Metastases, Calcification, Tomography X-ray computed, Incidence

## Abstract

**Objectives:**

To verify the incidence of calcified brain metastases (CBM), illustrating the different presentation patterns and histology of primary tumor.

**Methods:**

A series of 1002 consecutive brain computed tomography (CT) scans of patients with known primary tumors was retrospectively assessed. CBM were defined by the presence of calcification within intra-axial-enhancing lesions; identification of CBM was based on visual examination and ROI analysis (> 85 Hounsfield units). Also, calcifications in the primary tumor of all patients with brain metastases were evaluated. In CBM patients, we investigated the type of calcifications (punctate, nodular, cluster, ring, coarse), the histology of primary tumor, and if a previous RT was performed.

**Results:**

Among 190 (18.9%) patients with brain metastatic disease, 34 presented with CBM (17.9%). Sixteen patients were previously treated with RT, while 18 presented calcifications ab initio (9.5% of all brain metastases). The majority of patients with CBM had a primitive lung adenocarcinoma (56%), followed by breast ductal invasive carcinoma (20%) and small cell lung carcinoma (11.8%). CBM were single in 44.1% of patients and multiple in 55.9%. With regard to the type of calcifications, the majority of CBM were punctate, without specific correlations between calcification type and histology of primary tumor. No patients with ab initio CBM had calcifications in primary tumor.

**Conclusion:**

In conclusion, our data show that CBM are more common than usually thought, showing an incidence of 9.5% ab initio in patients with brain metastases. This study underlines that neuroradiologists should not overlook intraparenchymal brain calcifications, especially in oncologic patients.

**Key Points:**

*• Among the differential diagnosis of brain intraparenchymal calcifications, metastases are considered uncommon and found predominantly in patients treated with radiotherapy (RT).*

*• Our data show that CBM are more common than usually thought, showing an incidence of 9.5% ab initio in patients with brain metastases.*

*• A proportion of intraparenchymal brain calcifications, especially in oncologic patients, might represent evolving lesions and neuroradiologists should not overlook them to avoid a delay in diagnosis and treatment.*

## Introduction

Intraparenchymal brain calcifications are found in a variety of conditions including physiological/age-related changes, infections, genetic and neurodegenerative diseases, vascular syndromes, metabolic/endocrine disorders, and primary tumors such as oligodendroglioma. Though in many cases calcifications can be considered an incidental finding, sometimes their presence can be crucial in making a correct diagnosis [1].

Brain metastases represent the most frequent brain neoplastic lesions in the adult population and their frequency has grown over time due to the increase in overall survival of oncologic patients and to the improvement of the available diagnostic tools for metastases detection, such as new-generation CT scanners and MRI [2]. Nonetheless, the occurrence of calcifications within brain metastases is uncommon and found predominantly in patients treated with RT [2–4]. Although some cases of calcified brain metastasis (CBM) are reported in the scientific literature, recent large series of cancer patients with CBM are not available and their real incidence has not been clearly defined.

Therefore, in the differential diagnosis of brain calcifications, CBM can be underestimated with the consequent risk of misdiagnosis and delayed treatment of the disease, especially when the presence of calcifications is observed ab initio (i.e., in absence of a previous RT).

The primary tumors reported to be responsible for CBM are squamous cell carcinoma and adenocarcinoma of the lung, breast adenocarcinoma, sarcoma of the mediastinum, squamous cell carcinoma of the cervix, adenocarcinoma of the pancreas, non-Hodgkin’s lymphoma, osteosarcomas, and colorectal and ovarian adenocarcinomas [5–10].

The primary purpose of our study was to evaluate the incidence of CBM considering a large population of patients with known primary tumors who underwent brain CT for staging. The second aim was to investigate which types of primary tumors were more commonly associated to CBM and whether CBM presented ab initio or post-RT. We finally investigated the presence of calcification in the primitive tumors of patients with brain metastases.

## Materials and methods

All patients signed an informed consent for the computed tomography (CT) scan and the requirement for an informed consent for the purpose of this study was waived by the institutional review board.

### Patients

This study retrospectively evaluated the brain CT scans of 1002 patients with known primary tumors afferent to the Oncologic Department of our Hospital. The examinations were performed in our Institution from October 2015 to October 2018. The clinical questions to perform a brain CT scan were carried forward for staging/restaging, for a clinical suspicion of brain metastases in patients with a recent diagnosis of primary tumors and for follow-up purposes.

### CT imaging analysis

The CT scans were obtained using a GE Lightspeed 32 slice CT scanner. Each CT was performed before and after the injection of a iodinate contrast medium (Iopamiro® 370, 1.5 ml pro Kilo).

Two radiology residents (N.R. and G.R.) with 4 and 3 years of CT experience, respectively, analyzed the brain CT scans of the patients and preselected those in which cerebral intra-axial metastases were present. Moreover, the presence of calcification in the primary tumor of patients with brain metastases and the presence of extracerebral metastases in all patients were evaluated by another radiology resident with 2 years of CT experience (G.S.) and reviewed by a certified radiologist (L.S.). Subsequently, two radiologists (L.S. and L.R.), with more than 10 years’ experience, analyzed the images of patients with brain metastases and selected those with evidence of CBM.

CBM were defined by the presence of calcification within intra-axial enhancing lesions.

In most cases, calcified lesions were observed de novo (i.e., new calcified lesions, demonstrated comparing the CT examination with the previous one in which they were not present). Furthermore, a possible different nature of calcifications was assessed by appropriate CT follow-up imaging and MRI when needed.

Calcifications were visually identified and only hyperdense areas with a value ≥ 85 Hounsfield Units (HU) were included to prevent the inclusion of intralesional hemorrhage [10, 11].

Small ROIs (area of 5 mm^2^ maximum) were drawn, on pre-contrast CT images, in the most hyperdense part of the calcific lesion with a special attention to exclude the peripheral part of the calcification in order to avoid the inclusion of intralesional non-calcific regions or normal brain parenchyma. In all lesions, apart from those with punctate and very small nodular calcifications, multiple ROI measurements were performed, and the highest HU value was considered for inclusion/exclusion.

**Fig. 1:**
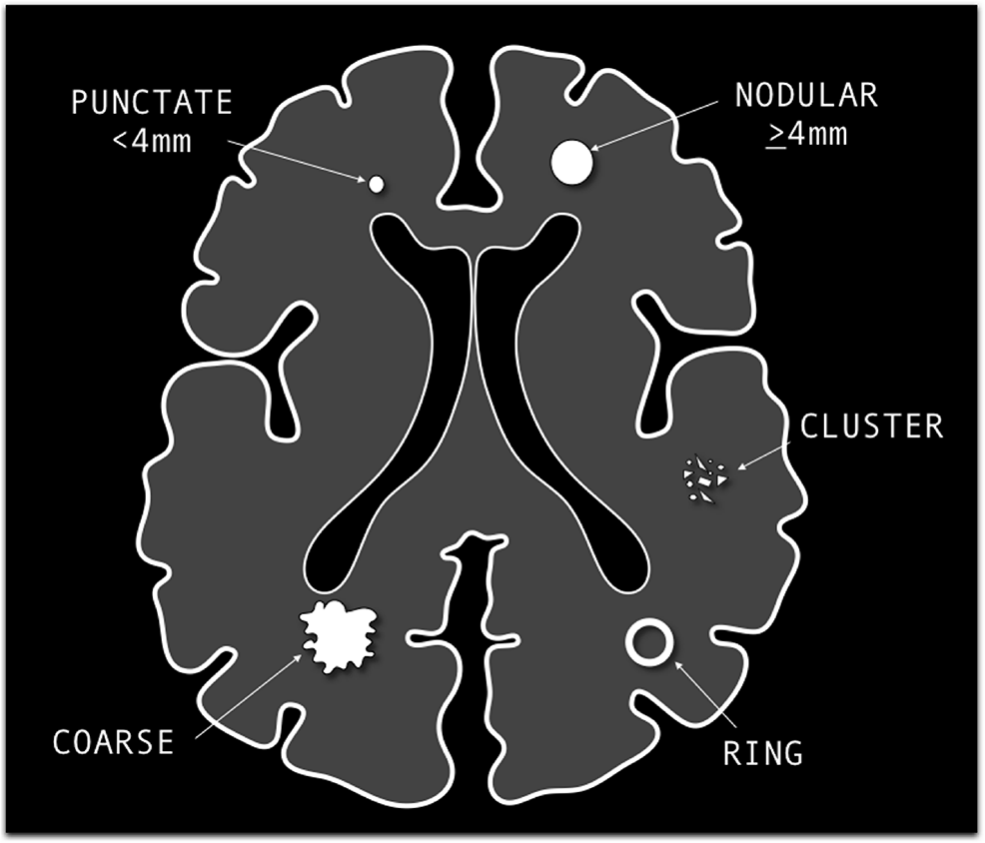
Schematic drawing of types of calcification found in CBM

**Fig. 2:**
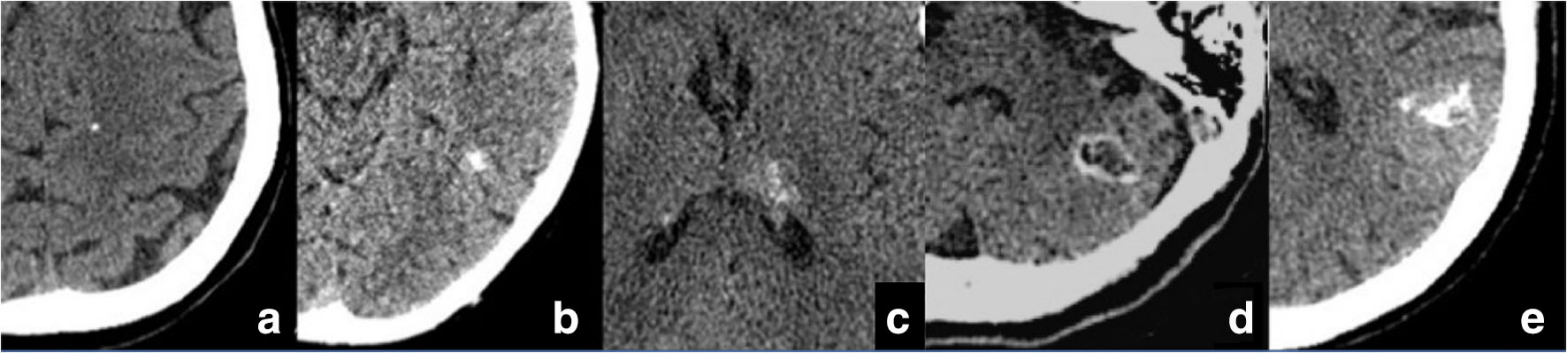
Types of calcifications (pre-contrast CT scan): punctate (**a**), nodular (**b**), cluster (**c**), ring (**d**), and coarse (**e**)

Calcification types were annotated according to the following classification: punctate (< 4 mm), nodular (≥ 4 mm), ring, cluster, and coarse (Figs. 1 and 2). Moreover, for each patient with CBM, the number of CBM (single or multiple), the primary tumor histology, and the occurrence of a previous treatment with brain RT were reported. Also, we investigated the presence of calcifications in the primitive tumor of all patients with brain metastases, by careful evaluation of previous CT examinations for tumor staging and, when available, of mammographic images with respect to breast tumors.

With respect to post-RT CBM, the latency between the end of radiating treatment and the first CT scan with evidence of calcification in at least one metastatic lesion was calculated.

### Statistical analysis

Student’s *t* test for independent samples and chi-square test were used to compare respectively mean age and sex of patients with ab initio CBM and non-calcified brain metastases (NCBM) and between CBM (ab initio and post-RT) and NCBM. Fisher test was used to compare the frequency of calcification in the primary tumor of patients with and without CBM. The *p* value was considered significant when < 0.05.

## Results

Out of 1002 patients, there were 530 patients with metastatic disease (52.9%). In particular, there were 484 patients with only extracerebral metastatic disease (48.3%), 46 with isolated brain metastases, i.e., without extracerebral metastatic disease (4.6%), and 144 with both brain and extracerebral metastases (14.4%). One patient with a suspicious calcified lesion at CT was not included since the calcifications were demonstrated within a cavernous malformation, confirmed by MRI.

Thus, among 1002 patients, there was a total of 190 with brain metastases (18.9%).

Out of all the patients with brain metastases, the identified primary tumors were 118 pulmonary tumors (89 adenocarcinomas, 23 small cell carcinomas, 4 squamous carcinomas, 2 large cell neuroendocrine carcinoma), 33 breast tumors (33 invasive ductal carcinomas), 25 melanomas, 3 bladder tumors (urothelial type), 3 gastrointestinal tumor (adenocarcinomas), 2 prostate adenocarcinomas, 1 renal tumor (clear cell carcinoma), 1 ovarian mucinous carcinoma, 1 soft tissue sarcoma, 1 endometrial, 1 mesothelioma, and 1 Li-Fraumeni syndrome with breast, lung, and gastric tumors.

Among the 190 patients with evidence of intra-axial cerebral metastases, 34 patients presented with CBM (17.9%) and 156 patients presented with NCBM (82.1%). There was no statistically significant difference in age (*p* = 0.13) and sex (*p* = 0.16) between patients with CBM and NCBM.

**Fig. 3:**
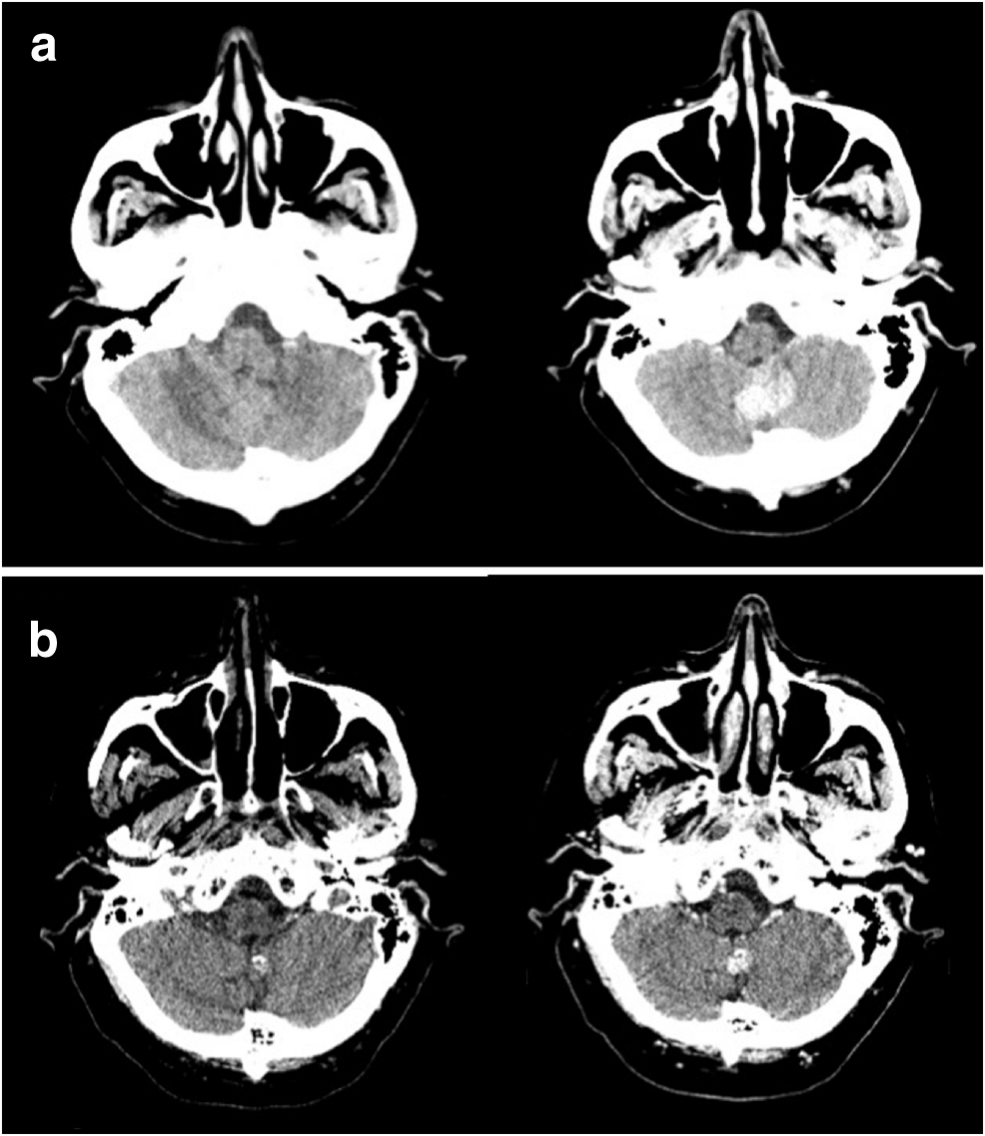
Post-RT CBM. Computed tomography, axial plane. **a** Pre- and post-contrast CT scan of a patient with a primary adenocarcinoma of the lung shows an inferior vermian metastatic lesion with intense enhancement and slight mass effect. **b** Follow-up CT scan of the same patient after stereotactic radiosurgery of the vermian lesion shows the appearance of a small nodular calcification within the lesion in the unenhanced CT, together with a definite reduction of the volume and of the associated mass effect

**Fig. 4:**
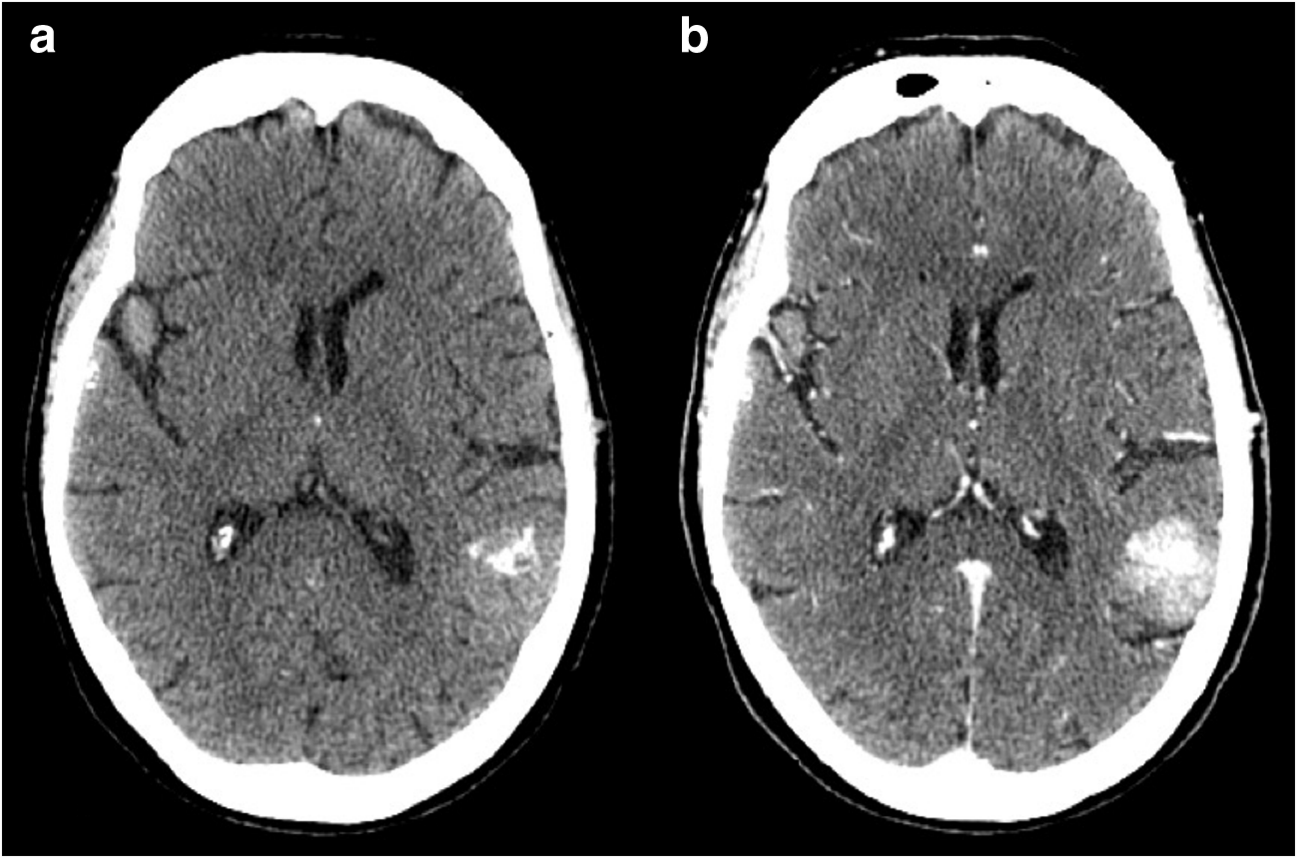
Ab initio CBM. Computed tomography, axial plane. **a** Pre-contrast CT scan with evidence of coarse brain calcification within a metastatic left temporoparietal intra-axial lesion, in a patient with a primary adenocarcinoma of the lung. **b** Post-contrast CT scan showing intense enhancement of the lesion. The patient, with multiple CBM (one of which can be partially seen in the right temporal lobe), did not undergo RT at the moment of diagnosis

Sixteen of the CBM patients underwent radiation therapy (either whole brain (WB), stereotactic radiosurgery (SRS), or fractionated RT on involved lesion (IFRT), according to radiation oncologist evaluation) before the appearance of calcification (Fig. 3), while the remaining eighteen patients presented with calcification ab initio **(**Fig. 4**)**. Therefore, 9.5% of all patients with brain metastases showed calcification ab initio within at least one metastatic lesion. Clinical and radiological characteristics of the patients are reported in Tables 1 and 2. In particular, Table 1 reports the latency time between the end of the radiating treatment and the first CT with appearance of calcification within at least one metastatic lesion. The mean time of calcification appearance was 78.8 days (SD 101.9; range 8–351).

**Table 1:** Patients with post-RT CBM

Sex	Age	Primitive	Number	HU	Type	Localization	RT	RT to CBM interval (days)
M	78	Adenocarcinoma of the lung	1	98	Ring	Left occipital lobe	SRS	23
F	73	Adenocarcinoma of the lung	2	85	Punctate	Vermis; left frontal lobe	SRS	287
F	61	Adenocarcinoma of the lung	2	87–102	Nodular	Right frontal and parietal lobe	SRS	184
F	48	Adenocarcinoma of the lung	1	100	Cluster	Right frontal lobe	SRS + WB	13
F	80	Adenocarcinoma of the lung	2	170	Nodular; ring	Right frontal lobe; left occipital lobe	WB	37
M	59	Adenocarcinoma of the lung	Multiple	165	Punctate; cluster	Cerebellum; left frontal and parietal lobe	WB	28
F	69	Adenocarcinoma of the lung	1	100	Punctate	Left frontal lobe	WB	19
M	76	Adenocarcinoma of the lung	1	85	Punctate	Right parietal lobe	WB	35
M	60	Adenocarcinoma of the lung	Multiple	92–108	Punctate; cluster	Pons; left frontal and temporal lobe	WB	8
F	82	Adenocarcinoma of the lung	Multiple	90–195	Nodular	Supratentorial	WB	20
M	68	Small cell lung carcinoma	Multiple	100–131	Punctate	Cerebellum; frontal lobes	WB	16
F	59	Small cell lung carcinoma	1	109	Punctate	Left thalamus	WB	127
F	62	Breast	Multiple	149–500	Punctate; cluster	Cerebellum	WB	17
F	70	Breast	1	373	Coarse	Left frontal lobe	SRS	351
F	62	Breast	1	125	Punctate	Right occipital lobe	WB	55
F	63	Breast	3	99–172	Punctate; cluster	Left frontal lobe; right and left parietal lobe	WB	41

**Table 2:** Patients with ab initio CBM

Sex	Age	Primitive	Number	HU	Type	Localization
M	73	Adenocarcinoma of the lung	1	250	Ring	Cerebellum
F	76	Adenocarcinoma of the lung	4	148	Nodular	Left parietal lobe; left thalamus; right frontal lobe
F	72	Adenocarcinoma of the lung	1	260	Nodular	Right parietal lobe
F	73	Adenocarcinoma of the lung	5	90–113	Punctate	Right and left frontal lobe
M	79	Adenocarcinoma of the lung	3	70–115	Punctate	Left frontal and temporal lobe
F	67	Adenocarcinoma of the lung	5	99–262	Coarse	Supratentorial
M	66	Adenocarcinoma of the lung	1	85	Punctate	Left frontal lobe
F	60	Adenocarcinoma of the lung	4	93–113	Punctate	Right and left frontal lobe
M	71	Adenocarcinoma of the lung	2	106–134	Punctate	Left frontal lobe
F	74	Small cell lung carcinoma	2	96	Nodular; punctate	Right frontal lobe; left temporal lobe
F	74	Small cell lung carcinoma	1	133	Punctate	Cerebellum
M	73	Squamous cell carcinoma	1	96	Punctate	Left frontal lobe
M	60	Large cell neuroendocrine carcinoma	1	142	Nodular	Left frontal lobe
F	76	Breast	Multiple	108–265	Punctate; cluster	Right frontal lobe
F	68	Breast	Multiple	92–112	Punctate; nodular	Infra- and supratentorial
F	70	Breast	1	85	Cluster	Right parietal lobe
F	58	Li-Fraumeni	1	135	Nodular	Left temporal lobe
F	73	Ovarian mucinous carcinoma	2	85–95	Nodular	Left frontal and parietal lobe

There was no statistically significant difference in age (*p* = 0.08) and sex (*p* = 0.26) between patients with ab initio CBM and NCBM.

The majority of patients with CBM had a primitive lung adenocarcinoma (19/34, 56%), followed by breast ductal invasive carcinoma (7/34, 20%), small cell lung carcinoma (4/34, 11.8%) and by single cases of large cell neuroendocrine carcinoma (1/34, 2.9%), squamous cell carcinoma (1/34, 2.9%), ovarian mucinous carcinoma (1/34, 2.9%), and Li-Fraumeni syndrome (1/34, 2.9%). In this last patient, with multiple primitive cancers (breast, lung, and stomach), it was not possible to identify the primitive tumor responsible for the metastatic disease. In our series, no CBM originated from melanoma and from other less common primary tumors, such as sarcomas, genitourinary tumors, and tumors of the gastroenteric tract.

Regarding the primitive tumors, we observed CBM in 19 out of 89 (21.3%) patients with lung adenocarcinoma (out of which 10/89 post-RT and 9/89 ab initio), in 4/23 (17.4%) of patients with small cell carcinoma of the lung (2/23 post-RT and 2/23 ab initio), in 7/33 (21.2%) patients with invasive ductal carcinoma of the breast (4/33 post-RT and 3/33 ab initio), in 1/4 (25%) of patients with squamous cell carcinoma of the lung (0/4 post-RT and 1/4 ab initio), in 1 patient with brain metastases ab initio from ovarian mucinous carcinoma, and in 1 patient with brain metastases ab initio from large cell neuroendocrine carcinoma.

With regard to the type of calcifications, the majority of CBM were punctate (14/18 patients with CBM ab initio and 10/16 patients with post-RT CMB), followed by the nodular and cluster types and by the far less common ring and coarse types.

CBM were single in 15/34 (44.1%) patients and multiple in 19/34 (55.9%). Eight patients out of 34 patients presented with an association of two different types of calcifications (3/18 in the ab initio group and 5/16 in the post-RT group). There were no specific correlations between calcification type and histological type of the primary tumor.

Out of 190 patients with brain metastases, CT scans or mammography were available for the assessment of calcification within the primary tumor in 180 patients. In 10 patients, 9 with breast ductal invasive carcinoma and 1 with lung adenocarcinoma, these examinations were not available.

Calcifications in primary tumors were found in 12/180 patients with brain metastases (6.7%).

Calcifications in primary tumors were 9/146 in patients without CBM and 3/34 in patients with CBM. The difference did not appear statistically significant (Fisher’s test = 0.70).

No patients with ab initio CBM had calcifications in the primary tumor while we found calcifications in 3 cases of breast ductal invasive carcinoma, in patients with post-RT CBM.

## Discussion

To the best of our knowledge, this is the study with the largest reported series of patients with CBM. Only a few case reports and small series of patients with CBM have been previously described. In 1976, Deck et al studied 57 patients with brain metastases and subdivided decreased density lesions from increased density lesions (without specifying their possibly calcific nature) [12]. In 1982, Anand and Potts collected 7 cases of CBM in a period of 8 months and described different types of calcifications: punctate, curvilinear, and amorphous [13]; in this series, the total number of analyzed patients is not reported. CBM are described in approximately 1% of surgical and 6.6% of autopsy specimens [10], while some authors citing the paper by Deck report an incidence of 3.5% of CBM found with CT [14]. The studies reported above are now outdated, especially considering the technological limitations of the available CT scanners. Identification of CBM is likely to be improved by newer CT technologies. In particular, multidetector CT scanners enable faster imaging with increased spatial and contrast resolution and reduced motion artifact, while dual energy CT has the potential to objectively differentiate calcific from hemorrhagic lesions by applying a material decomposition algorithm [15]. In our study, the threshold of 85 HU in association with visual evaluation has been considered to prevent the inclusion of intralesional hemorrhages which typically have a density in the range of 40–85 HU [11, 15, 16].

More recent studies report only single or limited number of cases of CBM [5, 7–10, 17–20].

In our series of 190 patients with brain metastases, we observed a frequency of 17.9% CBM (9.5% identified ab initio and 8.4% after RT). These results suggest that CBM occur more often than previously reported. In particular, with respect to the proportion of ab initio CBM, brain calcifications in CT scans should draw special attention, notably in oncologic patients. From an epidemiological perspective, ab initio CBM represent the most interesting group in our series, although the physiopathological mechanisms underlying their formation and eventual progression are not clear.

Identification of calcium density on CT lacks details regarding the chemical composition of calcified portions of CBM. We thus are unable to precisely characterize what type of calcification is present in metastatic lesions ab initio and in other calcified benign lesions.

Moreover, uncertainty regarding the precise biochemical composition limits speculations on the mechanisms leading to calcification formation. Recent studies in microclacifications in breast cancer suggest that calcifications are not simply the result of degenerative processes but results from complex cellular mechanisms which interestingly are also linked to metastatic capabilities of tumor cells. In particular, expression of osteopontin seems to regulate both cellular microcalcification and cancer cell migration [21].

Since the first report by Harwood-Nash et al [22], calcifications are considered one of the most common radiological manifestations of radiation-related brain injury. Also, the pathogenetic mechanism of calcifications developing after RT is not completely clear. The effect of RT causes hypoxia of the treated tissue, which determines a breakdown of the normal calcium gradient across the cell membrane with a consequent accumulation of calcium inside the cell. Such high concentration of calcium in the intracellular space seems to be the basis of calcification formation [23]. The calcification process, which seems to follow cell death, later extends to the extracellular space [17]. There is no clear association with the applied dose or fraction of the RT treatment, neither with the possibly concomitant systemic chemotherapeutic treatment [18].

Systemic chemotherapy could also influence the calcification process, but the mechanism has not been described and no specific treatment has been clearly indicated as responsible [18].

One study reported the disappearance of intratumoral calcifications in two patients with cerebral glioma followed by progression of the lesion [23]. In this report, the authors speculate that there could be relative stability of the tumoral microenvironment in the presence of calcium deposits.

Hwang et al reported the case of a patient with multiple CBM that did not undergo cranial RT, nor any further therapy, with an unusually prolonged survival of 2 years and 9 months from the time of initial demonstration of the brain lesions [19]. Another study describes a large cerebellar ab initio CBM that, due to slow progression, was treated 2 years after initial diagnosis [5].

Our study is not able to demonstrate a correlation between calcifications within cerebral metastases and the behavior of the lesions; thus, we cannot assert that ab initio CBM are more stable than non-calcific metastatic lesions.

In our series, CBM originated predominantly from primary adenocarcinomas of the lung, breast adenocarcinomas, and small cell carcinoma of the lung, while none originated from melanoma. The heterogeneous distribution of CBM in some patients, in which not all metastases have calcifications, is not clear. With respect to calcifications in primary tumors, our study has not identified a significant correlation between calcifications within the primary tumor and CBM.

Our work has limitations. Firstly, the inclusion of patients might be biased by the types of tumors most frequently afferent to our Institution, which partially reflect epidemiological data, being breast and lung adenocarcinomas the commonest primary tumors in the population. On the other hand, the small number of patients with less frequent tumors reaching our Institution, such as osteosarcoma, may have limited our results. Further studies could include a higher heterogeneity of primitive tumors.

Secondly, we were unable to correlate CT findings with MRI, which should be today the method of choice to study brain metastases [24]. Nevertheless, CT is a valid technique for the detection of intraparenchymal calcifications, although not the first choice in the work-up of patients with brain metastases which are optimally studied by MRI [24]. Notwithstanding the widespread use of susceptibility-weighted imaging (SWI) in MRI protocols, CT seems to be superior in the detection and characterization of intraparenchymal calcifications [3] and we do not know if careful examination of phase images, which should enable to discriminate blood degradation products from calcium [25], would yield the same results we obtained with the analysis of CT, particularly with small calcifications. Future studies could address this issue.

In conclusion, our data show that CBM are more common than usually thought, showing an incidence of 9.5% ab initio in patients with brain metastases. This study underlines that neuroradiologists should not overlook intraparenchymal brain calcifications on brain CT because they may represent evolving lesions in oncologic patients.

## Abbreviations

CBM: Calcified brain metastases
CT: Computed tomography
HU: Hounsfield units
IFRT: Fractionated radiotherapy
MRI: Magnetic resonance imaging
NCBM: Non-calcified brain metastases
RT: Radiotherapy
SRS: Stereotactic radiosurgery
SWI: Susceptibility-weighted imaging
WB: Whole brain
